# A decade of tuberculosis eradication programs in the Mediterranean water buffalo (*Bubalus bubalis*) in South Italy: Are we heading toward eradication?

**DOI:** 10.3389/fvets.2024.1405416

**Published:** 2024-07-26

**Authors:** Alessandra Martucciello, Maria Ottaiano, Piera Mazzone, Nicoletta Vitale, Anna Donniacuo, Roberta Brunetti, Marcellino Di Franco, Pasquale Cerrone, Claudia Spoleto, Giorgio Galiero, Esterina De Carlo

**Affiliations:** ^1^National Reference Centre for Hygiene and Technology of Breeding and Buffalo Production, Istituto Zooprofilattico Sperimentale del Mezzogiorno, Salerno, Italy; ^2^Istituto Zooprofilattico Sperimentale dell'Umbria e delle Marche “Togo Rosati”, Perugia, Italy; ^3^Istituto Zooprofilattico Sperimentale Piemonte, Liguria e Valle D’Aosta, Turin, Italy; ^4^Azienda Sanitaria Locale, Caserta, Italy; ^5^Azienda Sanitaria Locale, Salerno, Italy

**Keywords:** tuberculosis, water buffalo, *Bubalus bubalis*, diagnosis, gamma-interferon test, intradermal test, eradication, epidemiology

## Abstract

The water buffalo (*Bubalus bubalis*) is susceptible to bovine tuberculosis (TB), which receives increased attention in areas where buffalo breeding is prevalent, such as in Southern Italy, especially in the Campania region, where 70% of the buffalo stock is bred. Since 2012, TB testing in buffalo herds has been conducted using the Single Intradermal Test (SIT), with the Comparative Intradermal test (CIT) used in cases of inconclusive results. From 2012 to 2016, the interferon-gamma (IFN-γ) test was occasionally employed experimentally in herds with TB outbreaks to expedite eradication efforts. A local TB eradication program was implemented in officially TB-free buffalo herds between 2017 and 2019. This program involves initial screening with SIT, followed by confirmatory tests, including CIT and IFN-γ, for positive reactions. Since June 2019, the IFN-γ test has replaced the CIT in officially TB-free herds upon positive SIT reactions. Additionally, in suspected and confirmed TB-outbreak herds, the IFN-γ test was used at the discretion of the competent authority. Between 2017 and 2019, approximately 295,000 buffaloes in Campania were screened annually with *in vivo* tests provided by TB eradication programs. During this period, 32,040 animals from 855 herds were tested using the IFN-γ test and 4,895 tested positive. Since 2020, the use of IFN-γ testing has increased, and has become a prerequisite for the acquisition of TB-free status and is being systematically applied for TB outbreak-extinction procedures. The test was performed in all breeding buffaloes in cases of doubtful SIT results in TB-free herds and when TB lesions are detected at slaughter in animals from TB-free herds. This combined approach helped detect more TB outbreaks, and thereby led to a reduction in the TB prevalence and incidence rates. By 2022, the prevalence had decreased to 1.56%, and the incidence had decreased to 0.73%, after the increased use of the IFN-γ test. This study highlights the effectiveness of implemented strategies in reducing TB in this region. Overall, the data demonstrate the successful impact of TB eradication measures and surveillance activities in reducing bubaline TB prevalence and incidence in the Campania region.

## 1 Introduction

Bovine tuberculosis (TB), caused by a *Mycobacterium tuberculosis* complex (MTBC) infection, primarily *M. bovis* and *M. caprae*, affects diverse hosts, including some livestock species, wild animals and humans (1). The TB-susceptibility of the water buffalo (*Bubalus bubalis*) (2–4) has garnered attention in areas where buffalo breeding is highly developed. Approximately 70% of Italian buffalo herds are raised in Southern Italy, particularly in the Campania Region where, in 2023, the buffalo population was estimated to comprise 1,212 herds and a total of 305,023 animals raised. According to the Italian National Livestock Database (5), approximately 61% of the Campania buffalo herds are in the province of Caserta, wherein buffalo is an important livestock resource for the production of buffalo-milk-derived mozzarella (6). With an estimated revenue of EUR 766 million in 2019, this sector contributes approximately 20% of the gross domestic product of the Campania region.

As this important economic resource for the agri-food sector in Campania is endangered by bubaline TB, the implementation of a TB eradication program in the last decade has provided an accurate diagnostic approach to rapidly detect TB outbreaks. Historically, TB eradication programs have been based on the “test and cull” strategy as well as slaughterhouse-based surveillance. For *in vivo* diagnosis, the methods used to detect MTBC infections rely on the measurement of the cell-mediated immune response (CMI) to mycobacterial antigens (7) through tests such as the single intradermal tuberculin test (SIT), the comparative intradermal tuberculin test (CIT) (8, 9) and, in recent years, the gamma-interferon release assay (IGRA) (7, 10, 11). To improve the effectiveness of TB surveillance and diagnostic activity in buffaloes, the Italian Ministry of Health and the Health Department of the Campania region have funded several research projects proposed by the National Reference Center for Hygiene and Technology of Breeding and Buffalo Production (CReNBuf).

The aim of the first project undertaken in 2012 (IZSME 02/2012 RC) (12) was to study and validate alternative methods for the *in vivo* diagnosis of buffalo tuberculosis. The Research Project “Testing and standardization of tests: gamma-interferon and ELISA,” was conducted to assess the feasibility of the use of the gamma-interferon (IFN-γ) test for the early diagnosis of TB in buffaloes. In this project, the IFN-γ test was used for the first time experimentally in the Mediterranean buffalo (11). Based on the results of the study, the test was introduced into bubaline TB eradication programs in the Campania region in the following years. According to a protocol approved by the Italian Ministry of Health and the Campania region, which mandated the simultaneous use of the IFN-γ test with SIT or CIT, initially, from 2012 to 2016, the IFN-γ test was used in buffalo herds that had experienced previous TB outbreaks. In 2015, additional studies (IZS ME 06/15 RC) (13) were conducted under the project “Study on the specificity of the intradermal test in the buffalo species (*Bubalus bubalis*): interfering microbial agents and involved immune mechanisms.” The project aimed to assess the presence of nonspecific reactivity in negative animals from Officially Tuberculosis-Free (OTF) herds and compare the specificity of SIT and CIT in the buffalo species, and thereby enrich the data that had already been acquired from previous studies on the use of the IFN-γ test (14).

With the encouraging results obtained from the abovementioned studies, since 2017, the IFN-γ test was used officially by including it in the regulations of the Campania region’s legislation (DD Campania n. 236/2016^1^; DD Campania n. 226/2016^2^) to complement the CIT in OTF herds. In 2019, the introduction of the Regional Regulation (DGRC 207/2019^3^) was a decisive turning point, and the IFN-γ test was used as an official primary or standalone test during TB outbreaks. During this period (2019–2021), in herds with suspected and confirmed TB-outbreak, the IFN-γ test was used at the discretion of the competent authority. Finally, in 2022, with the implementation of the Regional Regulation (DGRC 104/2022^4^), the IFN-γ test has become an official test that is an alternative to the SIT in TB outbreaks in order to accelerate activities for the extinction of TB outbreaks, and a negative result of the IFN-γ test has become a prerequisite for determining TB-free status. The IFN-γ test has been performed in cases of inconclusive SIT outcomes and for the detection of TB lesions at the slaughterhouse in animals from OTF herds. After the implementation of the new European “Animal Health Law,” Regulation (EU) 2016/429 (15), and Regulation (EU) 2020/689 (16), the IFN-γ test has been approved for use in buffalo transportation and trading.

Legislative efforts in the Campania region in the past decade has involved the addressal of issues associated with TB diagnosis by using a combined approach that involves *in vivo* tests for TB detection. Concurrently, great effort has been made to intensify disease control in slaughterhouses by enhancing passive surveillance activities.

Therefore, we aimed to verify the effectiveness of veterinary measures adopted to eradicate TB in buffaloes in the Campania region by evaluating the performance of the TB eradication program, from 2012 to 2022, in this region.

## 2 Materials and methods

### 2.1 Study population and data source

The Campania region (40°54′38″N 14°55′14″E) is located in Southern Italy, and is characterized by diverse landscapes and a significant presence of livestock in the agricultural sector, wherein traditional agricultural practices and livestock farming are an essential component of the local economy. Campania is famous for buffalo farming, particularly for breeding water buffaloes (*Bubalus bubalis*) for milk production, which is used to make the renowned mozzarella di bufala and plays a crucial role in the economic landscape of Campania.

According to the Italian National Livestock Database 2024 (5), most buffaloes are raised in the province of Caserta (185,379 buffaloes and 762 herds), which is located in the northern part of the region, and in Salerno (112,451 buffaloes and 406 herds).

**Figure 1:**
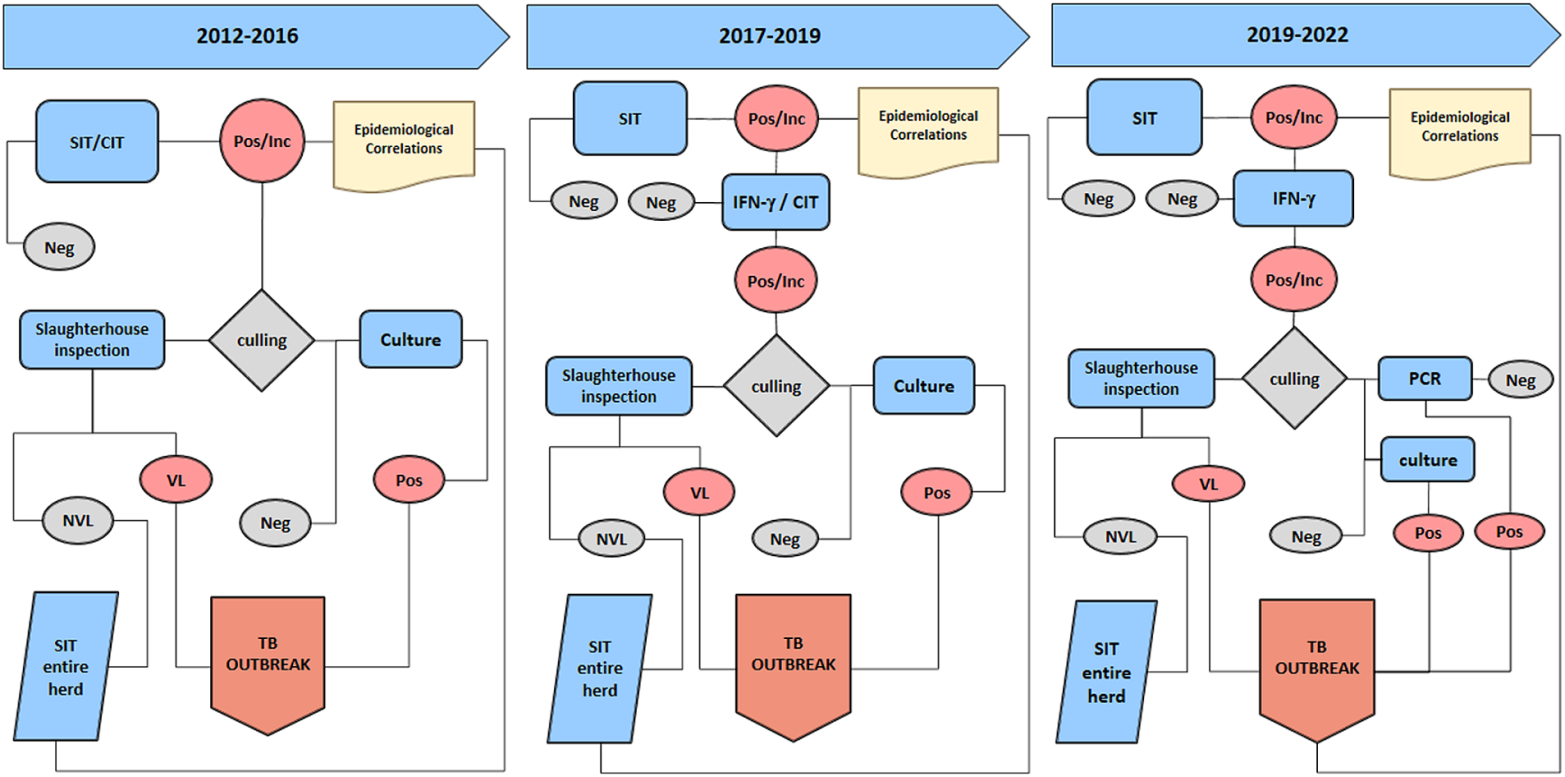
The diagram illustrates the diagnostic protocol and tests utilized after the three main regulatory modification. The first part displays the tests employed from 2012 to 2016, the middle section depicts the tests used from 2017 to 2019, and the right portion outlines the current Tuberculosis diagnostic protocol. SIT, Single Intradermal Test; CIT, Comparative Intradermal Test; Pos/Inc., positive inconclusive results; Neg, negative results, NVL, no visible lesion; VL, visible lesion.

**Figure 2:**
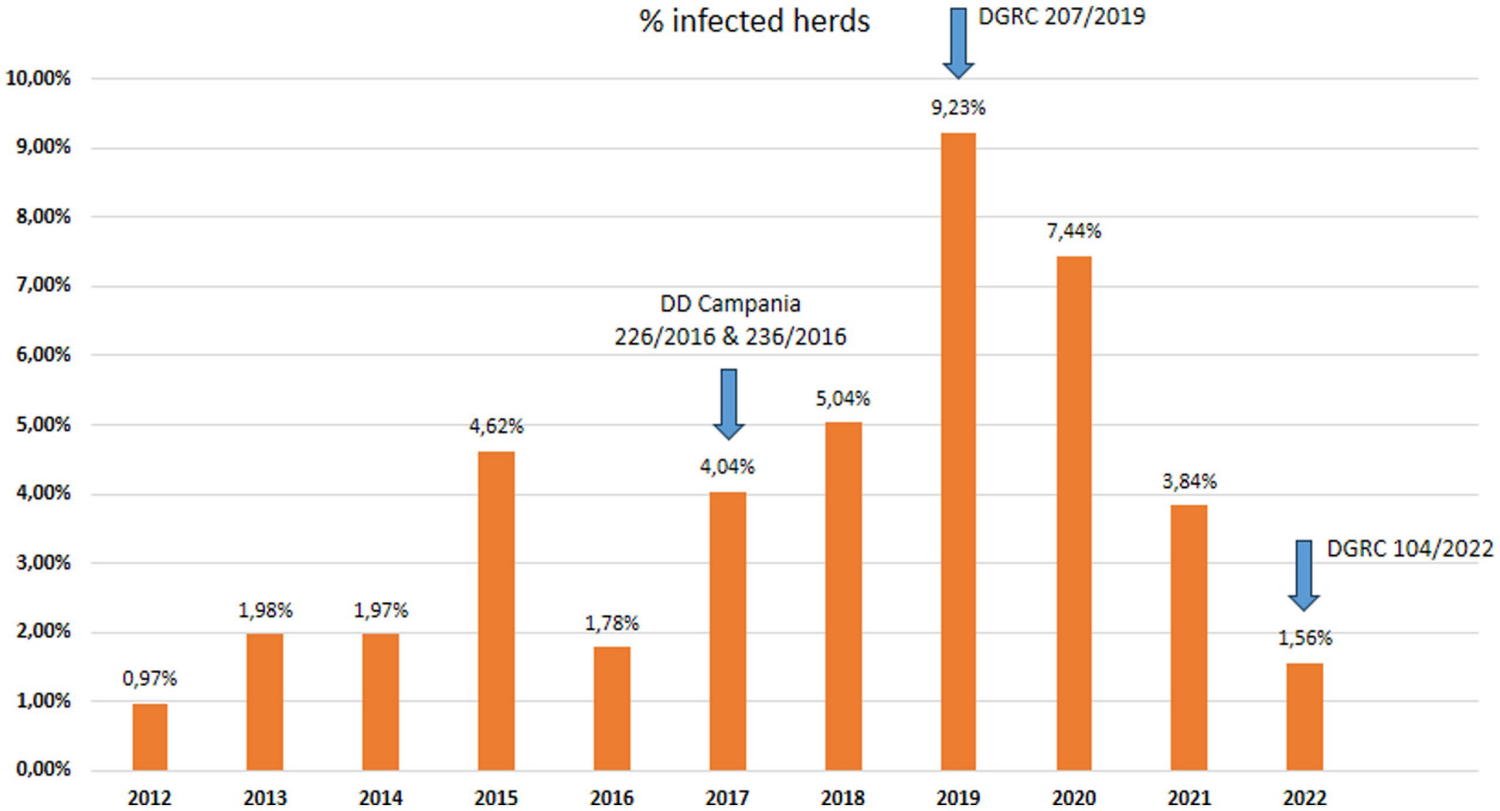
Herds prevalence: percentage of tuberculosis infected buffalo herds in Campania region in the period 2012–2022. The blue arrow shows the changes into eradication programmes introduced by regulation (data source Italian Ministry of Health SIR 2012–2022 – Vetinfo).

**Figure 3:**
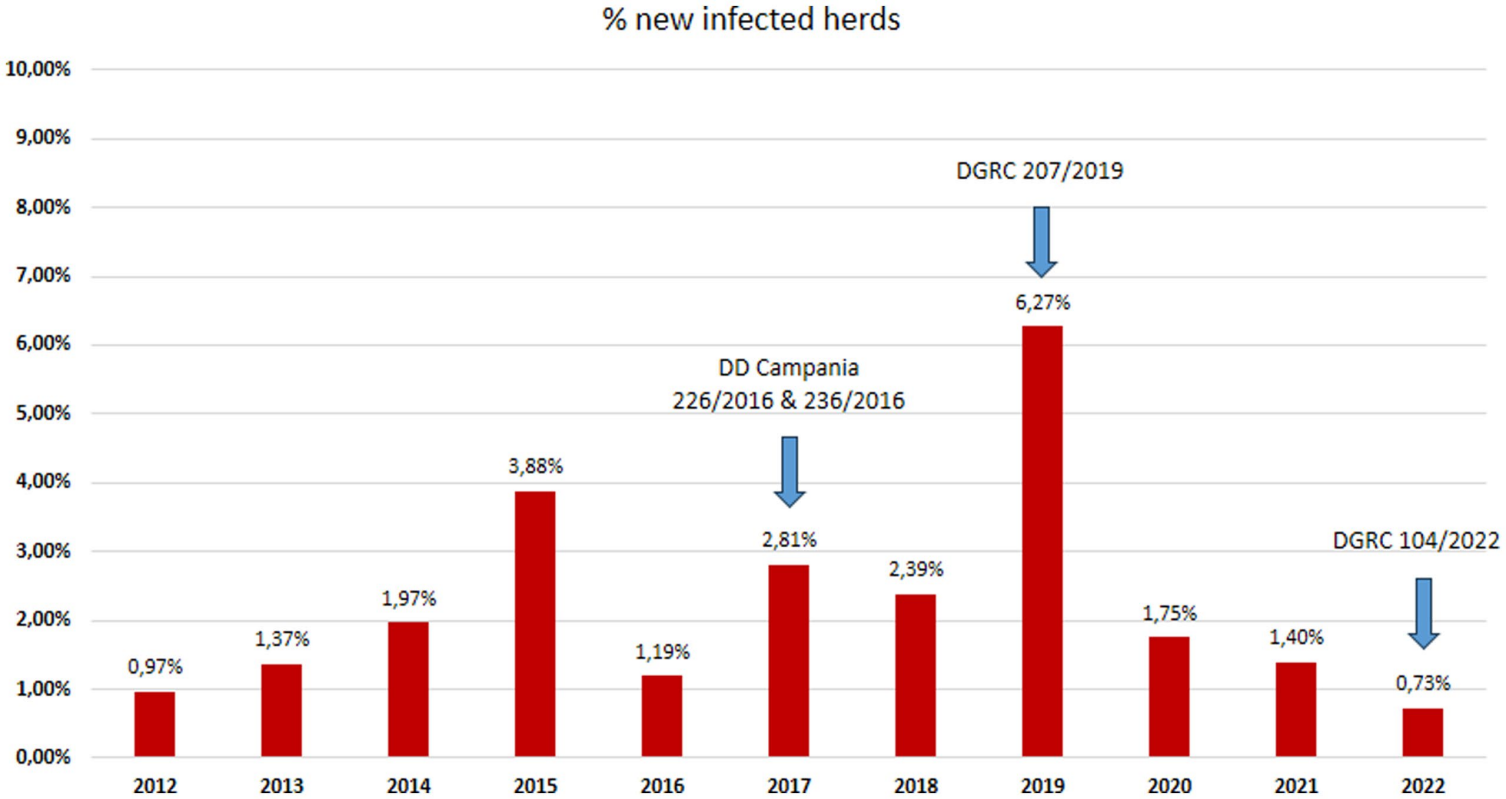
Herds incidence: percentage of new tuberculosis infected buffalo herds in Campania region in the period 2012–2022 (data source Italian Ministry of Health SIR 2012–2022 – Vetinfo).

The data used in the present study were collected within the context of the official TB eradication programme in accordance with European and Italian legislations (DM 592/95; DLvo 196/1999; Commission Regulation EC 1226/2002, O. M. 9 August 2012 and O. M. 28 May 2015 and subsequent amendments) (17–21) and regional regulations (DD Campania n. 236/2016; DD Campania n. 226/2016; DGRC 207/2019; DGRC 104/2022). The national and regional legislations applicable during the study period are shown in Supplementary Table S1 and Figures 1–3.

Data were extracted from the Italian National Veterinary Information Systems (5) and Laboratory Management System (SIGLA) of the Istituto Zooprofilattico Sperimentale del Mezzogiorno (IZSME). In particular, livestock data were obtained from the Italian National Database (BDN) and the animal health data were obtained from the Italian National Databases, and Animal Health Information System (SANAN); for notifiable disease outbreaks; moreover, data were extracted from the centralized notification System for Animal Infectious Disease (SIMAN) and the National Reporting Information System for Health and Economics to the European Commission for eradication, control, and surveillance plans subject to co-financing (SIR), all of which are accessible from the portal of the Veterinary Information System (5).

**Figure 4:**
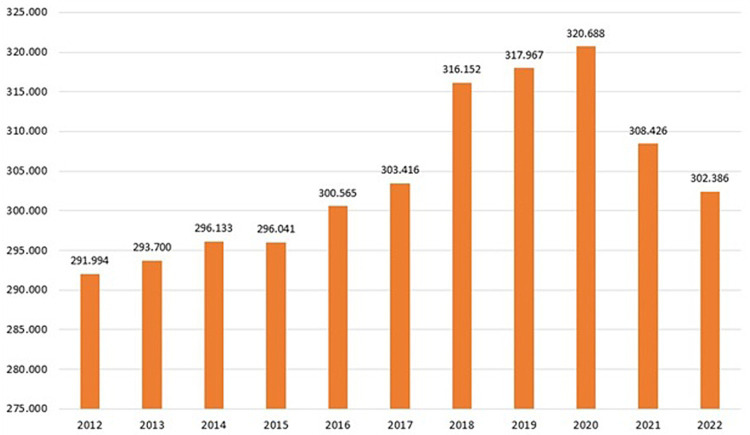
Distribution of the number of buffaloes in Campania Region for year (data provided by National Information System for health and economic reporting to the European Commission – SIR 2012–2022 Vetinfo).

**Figure 5:**
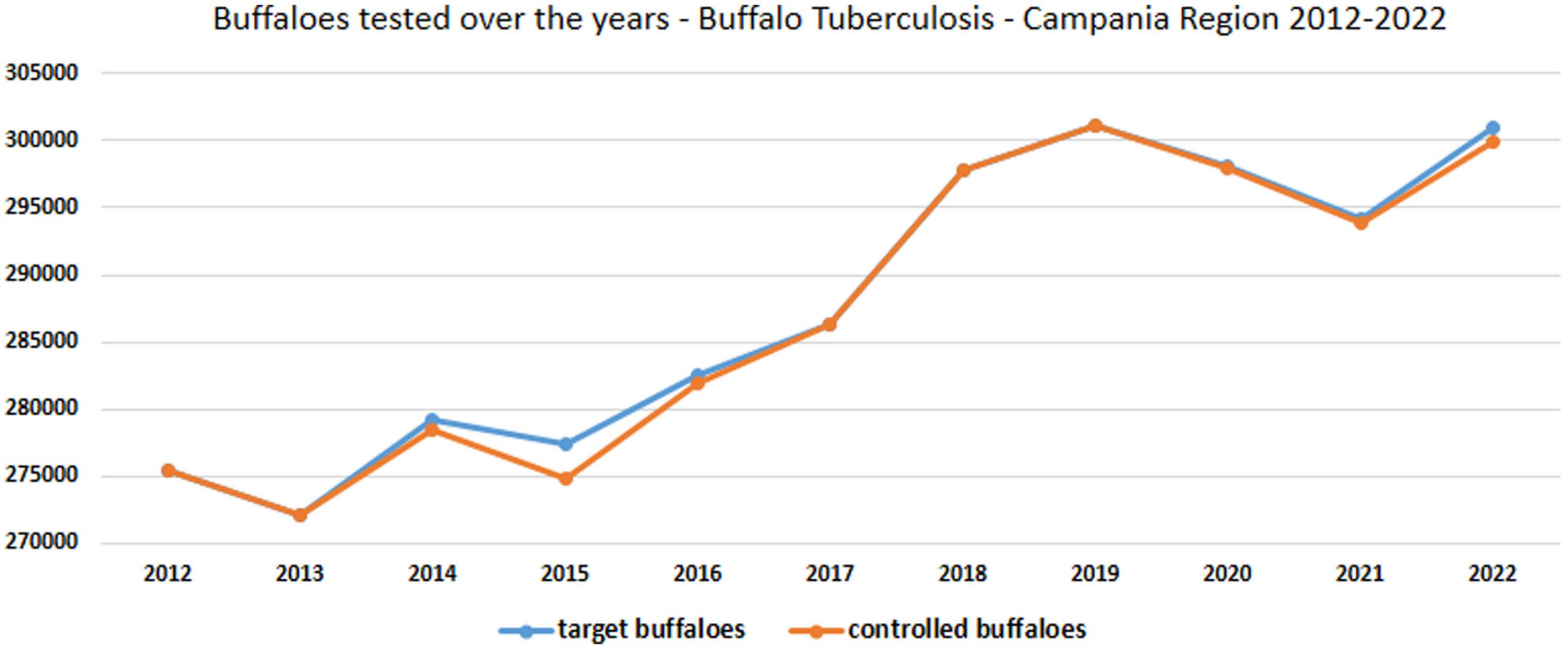
Trend of target buffalo population and buffaloes tested *in vivo* for tuberculosis from 2012 to 2022 in Campania region.

The analysis was conducted for all examined years (2012–2022). According to SIR (5), during that period, there were 291,994 to 320,688 buffaloes in the Campania region (Figure 4; Table 1), and nearly all of the buffaloes to be controlled under the TB eradication program were tested in the years examined (Supplementary Table S2; Figures 4, 5), except in 2014, 2015, and 2016 when approximately 1,000 buffaloes were not controlled. The regulation mandates the execution of the SIT for all individual buffaloes older than 6 weeks. As reported in Supplementary Table S1, in Italy, the national legislation in 2012 transposed an EU legislation (DM 592/1995; DLvo No. 196 of May 22, 1999 Implementation of Directive 97/12/EC amending and updating Directive 64/432/EEC on animal health problems affecting intra-community trade in bovine animals and swine; and O. M. 9/08/2012, O.M. 28/05/2015, and subsequent amendments) (17, 18, 20–23) provided TB control by SIT, and in cases of inconclusive results, enabled the possible use of CIT at the discretion of official veterinary services. During this period, the outbreak was initially confirmed based only on an intradermal test (DM 592/1995) (17). Later, with the transposition of the European legislation (Directive 97/12/EC amending and updating Directive 64/432/EEC) (24) with the Italian Legislative Decree of 196/1999 (18), confirmation of the outbreak required the isolation of *M. bovis* from the organs of slaughtered animals. According to Legislative Decree 196/1999 (18), a “suspected outbreak” commenced either after a positive SIT or following the detection of suspected TB lesions at the slaughterhouse. Therefore, in the Campania region, from 2012 to 2016, the SIT was used as an official test for the control of bubaline TB in all farms under the TB eradication program whereas the IFN-γ test has been used experimentally and occasionally in the TB outbreak to accelerate TB eradication procedures, in parallel with SIT after at least 42 days from the previous SIT. The IFN-γ test was used in research projects in an experimental protocol that was authorised by the Ministry of Health and the Campania Region (IZSME 02/2012 RC).

**Table 1:** Indicators of the TB eradication program in Campania, 2012–2022: herds and buffalo population, target population, number of tested herds/animals, coverage, infected herds and animals [number (percentage)], OTF/non-OTF herds [number (percentage)].

Year	Population		Target		Controlled		Coverage		Infected		OTF	Not OTF
	Herd	Buffaloes	Herd	Buffaloes	Herd	Buffaloes	Herd	Buffaloes	Herd	Buffaloes	Herd	Herd
2012	1,697	291,994	1,343	275,412	1,343	275,390	100.0%	99.99%	13 (0.97%)	868 (0.32%)	1,303 (97.0%)	27 (2.0%)
2013	1,639	293,700	1,316	272,087	1,316	272,087	100.0%	100.00%	26 (1.98%)	803 (0.30%)	1,280 (97.3%)	10 (0.8%)
2014	1,595	296,133	1,270	279,181	1,270	278,454	100.0%	99.74%	25 (1.97%)	855 (0.31%)	1,238 (97.5%)	7 (0.6%)
2015	1,537	296,041	1,212	277,413	1,212	274,877	100.0%	99.09%	56 (4.62%)	1,342 (0.49%)	1,153 (95.1%)	3 (0.2%)
2016	1,511	300,565	1,179	282,544	1,179	281,911	100.0%	99.78%	21 (1.78%)	259 (0.09%)	1,148 (97.4%)	10 (0.8%)
2017	1,447	303,416	1,140	286,315	1,140	286,315	100.0%	100.00%	46 (4.04%)	704 (0.25%)	1,076 (94.4%)	18 (1.6%)
2018	1,351	316,152	1,132	297,754	1,132	297,754	100.0%	100.00%	57 (5.04%)	6,015 (2.02%)	1,043 (92.1%)	32 (2.8%)
2019	1,326	317,967	1,116	301,032	1,116	301,032	100.0%	100.00%	103 (9.23%)	3,773 (1.25%)	980 (87.8%)	33 (3.0%)
2020	1,291	320,688	1,088	298,147	1,088	297,917	100.0%	99.92%	81 (7.44%)	2,913 (0.98%)	978 (89.9%)	29 (2.7%)
2021	1,277	308,426	1,069	294,081	1,069	293,885	100.0%	99.93%	41 (3.84%)	1,696 (0.58%)	1,015 (94.9%)	13 (1.2%)
2022	1,164	302,386	1,092	300,978	1,092	299,882	100.0%	99.64%	17 (1.56%)	532 (0.18%)	1,064 (97.4%)	11 (1.0%)

Data provided by the National Information System for Health and Economic Reporting to the European Commission, SIR Vetinfo.

Since 2017, the national and regional programs for buffalo TB eradication provided by the Ministry of Health and the Campania Region (DD Campania n. 236/2016; DD Campania n. 226/2016) prescribed the use of the SIT as a screening test, wherein positivity in OTF herds had to be confirmed, and the animals had to be retested. As confirmation tests, either the CIT or the IFN-γ test could be used 42 days after SIT. Culling was mandatory only in the case of CIT-positive buffaloes, whereas IFN-γ test-positive animals were slaughtered at the discretion of the competent authority in agreement with the breeders. Accordingly, in OTF herds with SIT positivity, health restrictions were imposed until further testing was performed (CIT or IFN-γ test) and the herd was designated as one with a “suspected TB outbreak.” If all confirmation tests yielded negative results, the herd regained the status of OTF. Conversely, if the confirmatory tests produced positive results, the animals were slaughtered; moreover, if there was a positive result in the culture examination for *M. bovis*, a TB outbreak was confirmed. Therefore, a confirmed TB outbreak was defined as a herd with positive SIT and CIT and/or a positive IFN-γ test confirmed by the isolation of *M. bovis* in at least one animal (Figure 1). According to the “test and cull” strategy, all animals that tested positive in any of the *in vivo* confirmation tests were culled.

Since 2019, with the introduction of DGRC 207/2019, the IFN-γ test replaced the CIT, which was then discontinued (Supplementary Table S1; Figure 1). With this regional regulation, control was intensified, and SIT was conducted annually in OTF herds. In high-risk areas (with a high-risk epidemiological situation and the presence of repeated outbreaks), control measures were carried out every 6 months.

Animals with a positive and/or inconclusive SIT screening test in the OTF herds underwent the IFN-γ test after 15 days, and the herd’s status was suspended; if all animals tested negative in supplementary investigations, the suspension was removed. Animals with positive SIT and IFN-γ test results were immediately slaughtered, and the herd’s status remained suspended. The animals underwent thorough postmortem examination at the slaughterhouse to verify the presence of TB lesions, and positive status because of the presence of TB lesions, positive *M. bovis* culture, or positive PCR confirmed the outbreak, and the herd’s status of OTF was downgraded from suspended to revoked. In confirmed TB outbreaks, the region, based on a proposal by the competent Local Health Authority (ASL), could authorize the use of the IFN-γ test for the rapid eradication of outbreaks, according to UE Regulation N. 1226/2002 (19). In 2022, with the implementation of DGRC 104/2022, the IFN-γ test became an official test for use in TB outbreaks as an alternative to the SIT in order to accelerate TB eradication activities to control TB outbreaks (Figure 1).

Simultaneously, a negative IFN-γ test result became a prerequisite for the acquisition or re-acquisition of TB-free status for OTF herds. Furthermore, in all breeding buffaloes in case of an inconclusive SIT in OTF herds, and with the detection at the slaughterhouse of TB-lesions in animals from OTF herds, the IFN-γ test was performed after 42 days from the SIT. Moreover, with the enforcement of the European Union Regulations EU 2016/429 (15) and EU 2020/689 (16) and in accordance with the Italian Legislative Decree of August 5, 2022 n. 134 (22), which transposed European regulations into Italian law, the IFN-γ test is used for validating animal transportation and trading.

Within the framework of the regional laws promulgated by the Campania region, in addition to those that defined the aspects of TB eradication and control, the regional law DD 59 of 03/03/2017^5^ concerning the approval of diagnostic procedures for the eradication of buffalo TB in Campania also dealt with aspects related to the field training of official veterinarians. In particular, the *in vitro* and postmortem procedures were harmonised, guidelines were defined, and on-farm and at-the-slaughterhouse coaching of official veterinarians was carried out by veterinarians from the IZSME and CReNBuf.

### 2.2 Intradermal skin test

In accordance with EU regulations and Italian legislation (DM 592/1995; DM 196/99, and the Ministerial Order of August 9, 2012) (17, 18, 20), intradermal tests were performed by the official veterinary services of the territory. The SIT was applied to the left shoulder of the animal near the acromion spina scapulae, and skin thickness was measured and recorded using a calliper, followed by intradermal inoculation with 0.1 mL Italian Bovine Purified Protein Derivatives (PPDB) produced at the Istituto Zooprofilattico Sperimentale dell’Umbria e delle Marche, Italy (23). During the CIT, 0.2 mL Italian Avian Purified Protein Derivatives (PPDA) was inoculated into the right shoulder. After 72 h, hypersensitivity was read and recorded in millimetres by remeasuring the skinfold thickness. The results were interpreted as previously described (11). The reactors were considered TB-positive whereas the test was considered inconclusive and negative if the difference between the PPDB and PPDA measurements was ≥4 and > 1 mm and < 4 and ≤ 1 mm, respectively.

### 2.3 IFN-γ test

The *in vitro* IFN-γ test first developed in Australia in the late 1980s (25) has been recommended by World Organization for Animal Health (WOAH) since 1996 in the Manual of Diagnostic Tests and Vaccines for Terrestrial Animals. In the past, many TB eradication programs have relied on the use of the IFN-γ test in parallel with the intradermal test to maximize the detection of infected animals. The test has been accepted as an ancillary test to intradermal test in the European Union since 2002 with the Council Directive 64/432/EEC, as amended by (EC) 1,226/2002 (19). However, without a unique and official interpretative criterion, different IFN-γ test interpretation criteria and protocols were adopted in different European countries and territories. Finally, since the implementation of Regulation (EU) 2016/429 (15), the IFN-γ test was performed as provided in Section 2 of Annex III of Regulation (EU) 2020/689 (16) and as defined in the unique and official protocol published for this purpose on the website of the European Union Reference Laboratory for Bovine Tuberculosis (EURL-TB) (26), in accordance with Article 6 of Regulation (EU) 2020/689 (16).

For the IFN-γ test, before the inoculation of the tuberculin, each animal is subjected to blood sampling in tubes with lithium heparin. According to the IFN-γ test protocol adopted from 2012 to 2020, blood samples were stimulated with Italian Avian and Bovine PPDs; the two PPDs were provided by Thermo-Fisher Scientific (Thermo-Fisher Scientific, Schlieren, Switzerland), pokeweed mitogen was used as a control for lymphocyte vitality, and phosphate buffer saline (PBS) was used as the Nil Control Antigen (NIL). To detect and quantify the release of the IFN-γ by lymphocytes, a sandwich enzyme-linked immunosorbent assay (ELISA) was used as per the manufacturer’s instructions (Bovigam, Thermo-Fisher Scientific, Schlieren, Switzerland). The criterion for the interpretation of the test results was previously reported by Martucciello et al. (11) and considered the agreement of the outcomes obtained with those of two pairs of PPDs. In cases without concordance between the outcomes of the two pairs of PPDs, the results were “inconclusive” and the animals were retested. During the experimental phase of the IFN-γ test, the ESAT6/CFP10 protein cocktail (27, 28) that was produced and purified, as described by Fontana et al. (29), at Istituto Zooprofilattico Sperimentale della Lombardia ed. Emilia Romagna was used to evaluate its possible utilization in the IFN-γ test to improve the test specificity (11).

Since 2021, according to the official IFN-γ test protocol used in the European community, European Standard Operating Procedures (SOP) of the EURL-TB (SOP/004/EURL), only a single pair of PPDs is to be used in the IFN-γ test, and the interpretive criterion provided by the EURL-TB is adopted (26, 30).

### 2.4 Slaughterhouse inspection and postmortem examination

All buffaloes that tested positive for infection in the antemortem tests were slaughtered and subjected to postmortem examination as stipulated by European and Italian legislation [DM 592/1995 and Commission Regulation (EC) No 1226/2002] (17, 19); target organs were collected (tonsils, retropharyngeal, mandibular, tracheobronchial, mediastinal, mesenteric, hepatic, sub-iliac, supra-mammary, popliteal, prescapular lymph nodes, lung, liver, and spleen) and sent to the laboratory for culture and biomolecular tests, as provided also by Regional law DD 59/2017. With the implementation of DGRC 207/2019, postmortem PCR testing was introduced as a confirmatory method for identifying a TB outbreak.

Briefly, the organs collected at the slaughterhouse were sent to the laboratory and processed within 24 h or frozen at −80°C. A portion of each sample was shredded and then transferred into a stomacher bag containing a physiological solution in a 1:2 ratio. The sample was then mechanically homogenized and divided into two aliquots, one for decontamination with a 4% NaOH solution for culture examination, and the other for PCR. The homogenate aliquot for PCR was inactivated by boiling, and then DNA was extracted from tissue samples through a mechanical lysis step, followed by a second step using the NucleoSpin Tissue Kit (Macherey-Nagel, Düren, Germany) according to the manufacturer’s instructions with a few modifications, as described by Ferrari et al. (31). MTBC detection was assessed directly in tissue samples using in-house *IS6110*-based real-time PCR, as previously described by Chiari et al. (32).

The culture examination was performed according to WOAH Terrestrial Manual protocols (33). Mycobacterial isolates were identified using molecular methods: multiplex PCR assay described by Kulski et al. (34), *gyrB* gene analysis by PCR-RFLP described by Boniotti et al. (35) for differentiation among *M. bovis*, *M. caprae*, *M. microti*, and human adapted MTBC species. The performance of the direct methods adopted, are described in the paper of Ferrari et al. (31), the PCR sensitivity was 91.84% (89.66–93.69% CI) and specificity was 95.24% (93.52–96.61% CI).

### 2.5 Statistical analysis

Baseline indicators, at the herd and animal levels, which were analysed to evaluate the performance of the TB eradication program in buffaloes in the Campania region are reported in tables and graphs. Specifically, for each year from 2012 to 2022, the number of tested herds/animals, coverage, prevalence, incidence, number and percentage of herds with different statuses (OTF/infected/non-OTF), number and percentage of slaughtered animals, number and proportion of herds with postmortem TB detection, and number and proportion of herds with reoccurrence were described. The coverage was calculated as the number of herds/animals tested divided by the number of herds/animals under the program. The prevalence was calculated as the number of positive animals/herds divided by the number of tested animals/herds, and incidence was calculated as the ratio of newly infected herds to OTF herds. The number of herds wherein TB was detected by postmortem inspection was calculated from the outbreaks revealed by slaughterhouse surveillance (SIMAN) (5).

An outbreak was considered a recurrent outbreak if a second episode of TB occurred at least 24 months after the outbreak was closed and culling measures were undertaken. From 2017 to 2020, other indicators were reported: the number and proportion of herds tested by the IFN-γ test, number and proportion of positives detected, and number and proportion of outbreaks detected by the IFN-γ test. These data were collected as part of ongoing research projects and experimental protocols authorized by the Italian Ministry of Health during the years considered.

To assess the effectiveness of eradication measures introduced with the regional program, the prevalence, incidence, and number of positives before and after the introduction of new legislation were compared by using Armitage’s chi-square test for trends, as we expected to observe an increase in all three indicators.

Finally, two maps of TB prevalence in the Campania region at the municipal level were produced to show the progression of the program: the plan in 2019, the year with the highest number of positive results, and the plan in 2022, which was the year with the lowest rate of positive results.

All data analyses were conducted using RStudio version 4.2.2, and QGIS3 software was used to produce the maps.

## 3 Results

Data on epidemiological indicators used to assess the effectiveness of the eradication measures that were adopted in the bubaline TB eradication programs in the Mediterranean buffalo in the Campania region in the years 2012–2022 were obtained from disease-eradication and surveillance activities, which were conducted both in the field and at the slaughterhouse.

**Figure 6:**
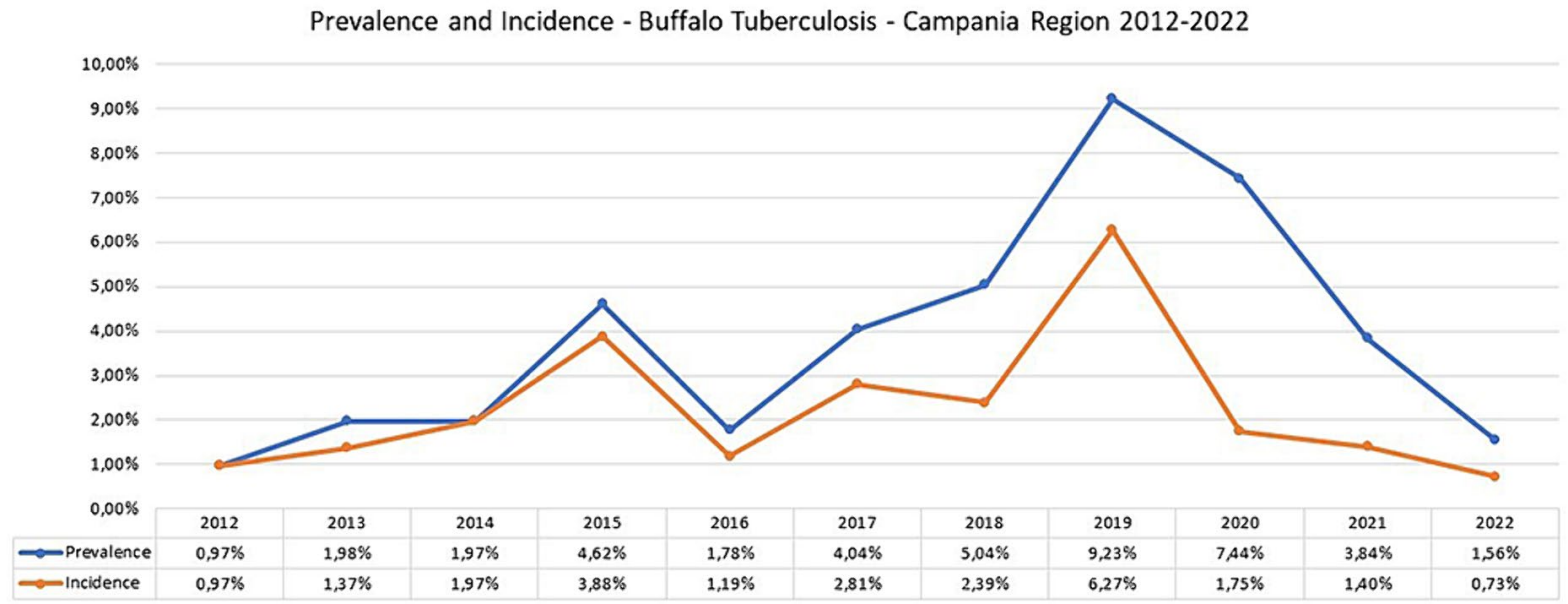
Prevalence and Incidence of bubaline tuberculosis in Campania region in the period 2012–2022 (data provided by National Information System for health and economic reporting to the European Commission—SIR Vetinfo).

The buffalo population in 2022 had slightly increased (302,386) since 2012 (291,994), whereas an overall reduction of farms was observed from 2012 (1697) to 2022 (1164). The number of controlled herds was 100%, which was consistent with the 100% legislation, whereas the number of animals was always higher than 99.6% (Table 1). A significant increase in the number of tested herds and buffaloes was observed between 2012 and 2015 (*p* < 0.005) and between 2016 and 2019 (*p* < 0.005) (Figure 5). As shown in Table 1, the number of outbreaks has fluctuated over the years. From 2012 to 2015, a steady increase in the number of outbreaks was observed, from 13 to 56. After a decrease in 2016, the number of outbreaks increased significantly from 2017 to 2019. The largest number of outbreaks was observed in 2019, with peaks in prevalence and incidence values (Figure 6). Spikes in prevalence and incidence were observed in relation to changes introduced in the eradication program after regional regulations were introduced in 2016, 2019, and 2022 (Figures 2, 3). Finally, a positive reduction in the number of outbreaks was registered in 2022 (*n* = 17) compared with the number of outbreaks registered in 2020 (*n* = 81).

In 2022, there were eight new TB buffalo herd incidents in Campania. This was the third consecutive year when the number of new TB buffalo herd incidents decreased in Campania, and was the lowest number since 2012. Between 2012 and 2015, the number of new TB buffalo herd incidents *per annum* had increased from 13 to 47. Following the introduction of the new protocol, the number of new incidents decreased in 2016, with 14 new TB herd incidents observed. Between 2017 and 2018, the total number of new incidents *per annum* was relatively stable between 28 and 32. In 2019, 70 new TB incidents were reported, which was the highest number observed during the entire period (Figure 6; Table 2). This reduction was driven by a marked decrease in the number of new TB herd incidents in the provinces of Caserta (8.3% reduction) and Salerno (3% reduction), as compared with previous years, and was statistically significant (*p* < 0.0001). The rate of increase in TB incidence decreased after peaking in 2019 (Figure 6). From 2012 to 2018, the incidence of TB showed a steady but significant upward trend (*p* = 0.005). The decreasing trend in 2019–2022 was significant (*p* = 0.0001), and the presence of TB seemed to be fading.

**Table 2:** Buffalo herds controlled with slaughterhouse surveillance and herds surveillance, TB-infected buffalo herds with partial or total eradication, and the annual number of recurrent TB outbreaks in the Campania region.

Year	Infected herds	Slaughterhouse surveillance	Herd surveillance	Newly infected herds	Infected herds, partial eradication	Infected herds, eradication	Recurrent outbreaks
2012	13	0	13	13	13	0	12
2013	26	1	25	18	24	2	15
2014	25	0	25	25	20	5	10
2015	56	0	56	47	55	1	14
2016	21	0	21	14	19	2	7
2017	46	14	32	32	46	0	10
2018	57	6	51	28	47	10	1
2019	103	22	81	70	90	13	1
2020	81	10	71	19	67	14	2
2021	41	9	32	15	38	3	0
2022	17	2	15	8	17	0	0

Data source: Italian Ministry of Health, SIR 2012–2022, Vetinfo.

The total culling in herds with a TB-outbreak before 2018 was low (Table 2). From 2012 to 2017, 10 total culling in TB-outbreaks were performed out of a total of 187 outbreaks, whereas the number of recurrent outbreaks was 58. After 2017, the total culling number increased to 40 of 299 outbreaks, and only four recurrent outbreaks were observed. The difference was statistically significant (chi-square test: 70.92, *p* < 0.00001). The introduction of the IFN-γ test, under the bubaline TB eradication program of the Campania region with an experimental protocol that was authorized by the Italian Ministry of Health, in 2017–2020, increased the sensitivity of the TB surveillance system, as the number of detected outbreaks increased from 2017 onward.

As shown in Table 3, a total of 855 herds were tested from 2017 to 2020 with the IFN-γ test and, in 105 herds, the test was able to detect infection; consequently, the number of infected animals culled increased.

**Table 3:** Herds and buffaloes controlled with the gamma-interferon test (IFN-γ) under the bubaline tuberculosis eradication program, Campania region, 2017–2020.

Years	Herds tested with IFN-γ	Buffaloes tested with IFN-γ	Herds IFN-γ+	Buffaloes IFN-γ+
2017	129	3,127	13	380
2018	240	3,782	18	1,437
2019	298	6,614	60	1,147
2020	188	18,517	14	1931
**Total**	**855**	**32,040**	**105**	**4,895**

After each change introduced by the new legislation (Figures 2, 3), in particular in 2017 and 2019, there was an increase in detected outbreaks, from 0.97% in 2012 to 9.23% in 2019. This trend was accompanied by a decrease in prevalence and incidence to 1.56% for prevalence and to 0.73% for incidence in 2022.

**Figure 7:**
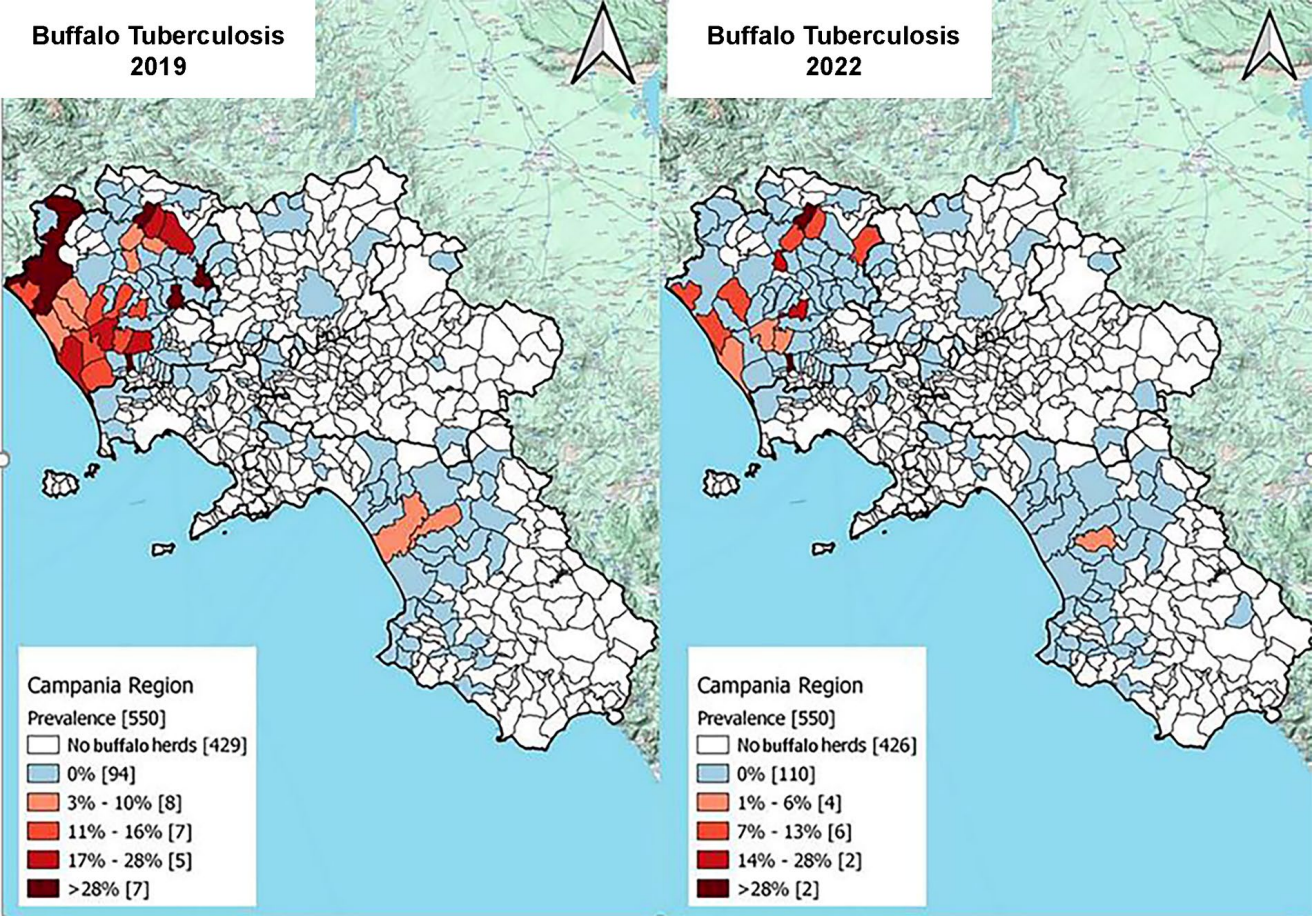
The maps show the distribution of municipality according to tuberculosis status. White polygons represent municipality without buffalo herds, blue polygons represent the municipality with only negative herd and finally, the red gradient the TB positive buffalo herds of Campania region in 2019 and 2022. The light red represent municipality with lower prevalence while darker red represent higher prevalence (data provided by the Italian Ministry of Health, Vetinfo SANAN 2019).

The Armitage’s chi-square test for trends was statistically significant for the prevalence (*p* < 0.001), incidence (*p* < 0.001), and number of positive animals (*p* < 0.0001) from before to after the introduction of the new legislation. The area with the highest concentration of buffalo herds and the highest TB prevalence was north of the province of Caserta (Table 4), from 2012 to 2022, and had the highest incidence. The distribution of municipalities according to the prevalence of TB in Campania is presented in Figure 7, which shows the years with the highest (2019) and the lowest (2022) TB prevalence.

**Table 4:** Incidence of bubaline tuberculosis in herds in the provinces of the Campania region, 2012–2022.

Province	2012	2013	2014	2015	2016	2017	2018	2019	2020	2021	2022
Avellino	0%	0%	0%	0%	0%	0%	0%	0%	0%	0%	0%
Benevento	0%	0%	0%	0%	0%	0%	0%	0%	0%	0%	0%
Caserta	1.24%	1.3%	2.34%	5.39%	1.78%	4.18%	3.45	9.21%	2.68%	2.03%	0.97%
Napoli	0%	0%	0%	0%	0%	0%	0%	0%	0%	0%	0%
Salerno	0.97%	0.25%	1.35%	1.1%	0%	0%	0.58%	0.58%	0%	0%	0.25%

Data source: SIR 2012–2022, Vetinfo.

## 4 Discussion

According to the European Food Safety Authority (EFSA) (36), in the past decade (2012–2021), the global occurrence of bovine TB in TB-infected areas was 1.3%, whereas in disease-free areas, it was 0.014% (36). In 2023, Bezos et al. (37) reported that, in 2021 in the EU, the prevalence of MTBC-infected cattle herds was very low (0.6%), and was only slightly higher than that in 2020 (0.4%). TB has been eradicated in many parts of Italy. The number of outbreaks recorded in 2021 (*n* = 138) was lower than those recorded in 2017 (*n* = 312). One-third of the outbreaks reported in Italy in 2021 occurred in the buffalo population in Campania, particularly in the Province of Caserta (*n* = 41). In Italy, the prevalence, excluding the buffalo population in Campania, as calculated by the National Reference Centre for TB for all of the country’s farms was 0.18% in 2021 and 0.34% in 2017. The Campania Region remains a non-TB-free area, as reported by the Italian Ministry of Health. In Italy, in 2022, a total of 127 TB outbreaks were reported in cattle, whereas in the buffalo population, 18 TB outbreaks were observed in the Campania Region, of which, 17 occurred in the Province of Caserta.

Between 2012 and 2015, the number of bubaline TB outbreaks increased from 13 to 56, and these were detected only by antemortem testing in the Campania region. During the same period, a high number of recurrent outbreaks, with a second episode of TB occurring at least 24 months after the eradication of the outbreak, was observed (Table 2), and thus there emerged a perception that the measures taken to eradicate TB outbreaks were inadequate, since TB outbreaks were recurring often in the same herds. In addition, some suspected outbreaks were not confirmed by either *in vivo* or postmortem examination, either by reporting at the slaughterhouse (suspected TB lesions) or with SIT positivity. Therefore, it was strongly suspected that the diagnostic sensitivity of TB eradication programs in the buffalo population might not be adequate to identify all TB outbreaks, and that the performance of the adopted diagnostic tools were weak and thus unable to detect the actual TB status in the Campania region. Therefore, since 2012, CReNBuf has proposed to the Italian Ministry of Health several research projects to improve and harmonize the *in vivo* diagnosis of TB in buffaloes (12, 13).

The strategic research activity focused on the evaluation of the use of the IFN-γ test for TB diagnosis in buffaloes. From 2012 to 2016, according to a protocol approved by the Italian Ministry of Health and the Campania region, which provided the use of the IFN-γ test in parallel with SIT or CIT, the IFN-γ test was introduced in buffalo herds that had previous TB outbreaks. In those years, the IFN-γ test proved to be more effective in detecting MTBC-infected animals in particular, and proved more reactive animals than did the SIT. Buffaloes that were TB-positive on the IFN-γ test were then confirmed to be infected through the finding of TB lesions at the slaughterhouse, and/or by positive culture test and/or PCR (11, 12).

In these years, specific Task Forces have been instituted (D.P. Reg. 04.08.2017, n.250^6^), involving the Campania regional authority, the CReNBuf and IZSME, the local University, and the Local Animal Health Authorities. Based on the encouraging research results obtained over the years, the IFN-γ test was used in the *in vivo* diagnosis of bubaline TB in the TB eradication programs through the Campania regional legislation (DD Campania n. 236/2016), wherein the use of the IFN-γ test alongside the CIT as a confirmatory test was recommended in subjects that had tested positive for SIT in the previous 42 days, in OTF herds.

With the implementation of the new regional regulations (Figures 2, 3), there has been a steady increase in both the percentage of herds identified as infected and the percentage of newly infected herds at 9.23 and 6.27% in 2019, respectively. The largest number of outbreaks was observed in 2019, with a peak in the prevalence and incidence values (Figure 6). Regarding the prevalence and incidence, spikes were evident with changes in diagnostic tools and control measures that were introduced in the TB eradication program after the implementation of the Regional Laws in 2016, 2017, 2019, and 2022 (Figures 2, 3). During the entire evaluation period, the number of outbreaks detected by antemortem surveillance based on the IFN-γ test and SIT was higher than that detected by postmortem surveillance performed at the slaughterhouse (Table 2). In particular, as shown in Table 3, a total of 855 herds were tested from 2017 to 2020 by the IFN-γ test and, in 105 herds, the test detected 4,895 TB-infected buffaloes. Following the detection of these TB-positive buffaloes in 2017–2019, the reactive animals were culled as per legislation (Supplementary Tables S1, S2; Figure 4). Furthermore, in some specific cases (herds with repeated outbreaks and a history of TB that persisted for years), it was decided to proceed with total culling (Supplementary Table S2). The total culling policy resulted in a drastic reduction in reinfections, which was found to be statistically significant (chi-square test 70.92, *p* < 0.00001). This type of action led to major problems for the breeders and stakeholders due to little or no understanding of the reasons for culling and, in some cases, there were even legal disputes against the Competent Authority. However, the policy of total culling that was adopted, when evaluated scientifically, proved effective and useful (Figure 6; Table 1). This measure resulted in significant financial losses for the Campania region. Approximately 6,750 and 5,220 animals were slaughtered in 2018 and 2019, respectively, as a result of the culling strategy adopted during the TB outbreaks (Supplementary Table S2). The cost of compensating buffalo farmers in the Campania region exceeded EUR 20 million in 2019.

The increased diagnostic sensitivity of the new approaches and tools introduced with DD 226/2016 justifies the high number of infected herds detected in those years. In 2019, 103 infected herds were detected, of which 81 were detected through herd surveillance and 22 were detected through slaughterhouse surveillance (Table 2). In those years, the Task Force analysed and discussed the results of the introduction of the new laws and, in 2019 the Campania region stated that the main cause of the increasing of prevalence, in that year, was due to the new Regional Decree (DD 226/2016) that provided the use of IFN-γ test, which proved to be more effective then SIT or CIT to find positive animals. Simultaneously, as reported by the IZS del Mezzogiorno, the lack of biosecurity in the farms present in specific areas of the Province of Caserta caused a high number of infected herds, with an incidence of 9.21% in 2019 (Tables 3, 4). This pattern is evident in Figures 2, 3, wherein after each change introduced by the new legislation, there was an increase in detected outbreaks, followed in subsequent years by a strong reduction in the prevalence and incidence, 1.56 and 0.73%, respectively, in 2022. The decrease was statistically significant for prevalence (*p* < 0.001), incidence (*p* < 0.001), and the number of positive animals (*p* < 0.0001) before and after the introduction of the new legislation. Therefore, in the last 2 years of the Campania bubaline TB eradication program, a reduction in the number of outbreaks was registered in 2022 (*n* = 17) in comparison with the number of outbreaks registered in 2020 (*n* = 81).

The TB eradication program actions adopted in Spain over the last ten years are similar to the measures introduced in the Campania region, particularly in high-risk areas. In Spain, after an increase in epidemiological indicators, especially in 2015 and 2016 (similarly in Campania region in the years 2017–2019), probably due to the increase in diagnostic performances in the field, a decreasing trend started in 2016, with a significant decrease of 19% in 2017 compared with 2016 (37).

The epidemiological indicators of the study period suggest that many TB outbreaks that the SIT had not detected in previous years were identified using the IFN-γ test. The incidence of TB in buffalo herds increased from 1.19% in 2016, 2.81% in 2017, and 2.39% in 2018, to a peak of 6.27% in 2019 after 3 years of TB control with the IFN-γ test, as introduced by new regional legislation (DD 226/2016) (Figures 5, 6).

In cattle and buffaloes (7–9, 38, 39), the sensitivity of the intradermal tests is subject to a large number of limitations related to: (i) the animal being tested (pre-allergic phase of TB infection, co-infection or exposure to an environmental mycobacterium, concurrent infection with immune system depressing viruses); (ii) the tuberculin used (use of a sub-potent product); (iii) the method of administration, reading and recording of the test (tester errors due to inexperience, lack of attention, poor cattle restraining facilities, poorly maintained testing equipment, etc.); and (iv) different epidemiological situations.

Regarding the SIT, a statistically significant association between the test outcome and the inoculation site in animals from TB-infected herds was reported, with higher probabilities of positive results when the test was performed in the anterior neck area (40). According to Casal et al. intradermal test sensitivity may be maximized by considering the area of the neck where the test is performed (40). Moreover, the intradermal test execution and interpretation can be affected by the differences in skin thickness between buffalo and cattle, by the black colour of the buffalo skin, and the harder tissue structure (11, 14). In fact, in the middle third of the cervical region, skin thickness ranges between 15 and 30 mm in buffalo vs. 5–8 mm in cattle (41).

In our experience, the IFN-γ test can overcome some of the limitations of intradermal tests. In a previous study conducted in Italy (11), the IFN-γ test in buffaloes could achieve high sensitivity and specificity values. In particular, using the test interpretation criterion recommended by the manufacturer, that is the same approved by the EU reference laboratories for bovine tuberculosis (SOP/004/EURL) (30), the diagnostic sensitivity of the IFN-γ test was 94.7% [Confidence Interval (CI) 95%, 92.3–96.5], and the specificity was 98.5% (CI 95%, 98.5–96.9). Thus, the IFN-γ test was useful for improving TB diagnostic strategies in buffalo herds (11), and this result is in agreement with that of other studies conducted in buffaloes (42, 43).

TB eradication is challenging because of several factors that include not only the limitations of antemortem diagnostic tests, but also the limitations of postmortem diagnosis (8). Postmortem inspection of slaughterhouses is one of the main surveillance measures for the detection of TB-infected animals in OTF countries. In non-OTF countries, postmortem inspection is an important surveillance tool to detect non-reactive TB-infected animals not detected by antemortem testing, which is normally provided in TB eradication programs (44). However, despite its relevance, post-mortem inspection has limited sensitivity (8, 45). TB-like lesions vary greatly in size and location, and may be missed during routine postmortem inspections at the slaughterhouse. Furthermore, the percentage of TB-infected animals detected at the slaughterhouse during post-mortem inspection depends on the accuracy and other circumstances related to the slaughterhouse (46, 47). Wide variation in the performance of surveillance and postmortem inspection in slaughterhouses has been described in Ireland, Great Britain, and Northern Ireland, where tuberculosis eradication reports were similar to what has been observed in the Campania region (48–51). The reported differences in slaughterhouse surveillance performance have been attributed to the animal and herd characteristics of the slaughtered population (e.g., geographical origin, production type, TB herd history, and animal age) or factors associated with the slaughterhouse itself (size, line speed, and veterinary inspector’s experience). Failure to detect infection at the slaughterhouse in animals from herds considered OTF, but actually TB infected, may contribute to the spread of TB in the herd and neighbouring herds through animal movements or contacts (44). Therefore, the implementation of postmortem inspection sensitivity is of great importance for the overall improvement of TB eradication/surveillance program performance.

With the aim of improving both *in vivo* and postmortem diagnostic tools, since 2017, the Campania region, together with the Istituto Zooprofilattico Sperimentale del Mezzogiorno and CReNBuf, have enhanced regional TB eradication programs through the implementation of mandatory training courses for official veterinarians involved in the execution of the program. As indicated by the Bovine TB Task Force (Task Force Subgroup DG SANTE) in the document “Working Document on Eradication of bovine TB in the EU” that establishes specific guidelines for Member States in order to accelerate the eradication process (SANCO/10,067/2013), from 2017, specific training was carried out, with the help of the Italian National TB Reference Centre (CRN TB) and CReNBuf. The training also included the procedures for carrying out diagnostic tests for TB (Campania region Decree DD 59 of 03/03/2017), to provide official veterinary services with an operational tool that was able to standardize procedures and harmonize operative methods for the eradication of TB in buffaloes. Therefore, veterinarians who perform intradermal TB tests, with particular reference to buffalo species, were also involved in using a system of audits to evaluate intradermal practices in herds. Courses were also conducted at slaughterhouses by experienced veterinarians who assisted slaughter inspectors during the postmortem examination of animals suspected of infection. According to EU and National regulations, official veterinarians have been obligated to perform sampling on all reactive animals considering lymph nodes with TB lesions but also lymph nodes of all anatomical districts (head, chest, abdomen, and limbs) in the absence of TB lesions. In addition, the epidemiological investigation introduced by the EU and Italian legislation (18) has provided considerable support in the management of outbreaks and areas at risk. In particular, in the Campania region with DD 226/2016, an epidemiological investigation was made mandatory within 2 days of the suspected outbreak of TB on the basis of the prevalence and risk of TB. Important information that can be acquired by epidemiological investigation includes the possible origin and duration of infection in the herd, and epidemiological links that require investigation (trace-back and trace-forward).

According to the current TB eradication program, each suspected TB-infected herd must be compulsorily subjected to a livestock movement cessation if not slaughtered, and all milk produced on the farm must be pasteurized before being processed (Supplementary Table S1). Herds should be subjected to a thorough epidemiological investigation based on questionnaires to identify the possible source of infection and indicate the suspected cause. Finally, the isolation of bacterial strains and genotyping are important tools for epidemiological control and disease eradication. This can provide information on the source of infection and identify risk factors for the spread and maintenance of tuberculosis (10). As is carried out in Spain also in Italy for the genotyping of MTBC strains (37), the CRN-TB receives all mycobacteria isolated from official territorial laboratories and carries out genotyping and molecular epidemiology support.

In Campania, several factors contributed to the improvement of epidemiological indicators, as confirmed by the low number of TB outbreaks in 2022. In fact, the rate of increase in TB incidence decreased, after peaking in 2019 (Figure 6). From 2012 to 2018, the incidence of TB showed a steady but significant upward trend (*p* = 0.005) due to improved diagnosis of TB both *in vivo* and postmortem, which allowed the detection of more TB outbreaks. The decreasing trend for the period 2019–2022 was significant (*p* = 0000.1), and the prevalence of TB now seems to be decreasing, this is likely due to the effectiveness of the measures taken to eradicate TB outbreaks (e.g., total culling strategy). Similar to what has been done in other countries such as Ireland and Spain to enhance TB eradication and control (37, 52), the key actions to reduce TB carried out in buffaloes in the Campania region included:

- the introduction of the gamma IFN test, which has significantly enhanced the *in vivo* diagnosis of TB in buffalo;
- the improvement of field diagnostic performance after the training courses and coaching implemented in 2017 for all veterinarians involved in the eradication programs, which resulted in a greater effectiveness of the on-site controls carried out by the official veterinary services of the Campania region, both for intradermal testing and for slaughterhouse inspection;
- the creation of the Task Force to strengthen TB management, involving the Campania regional authority, the CReNBuf and IZSME, the local University, and the Local Animal Health Authorities.

Other European countries have adopted similar approaches as that in the Campania region in 2012–2022. In particular, regarding the use of the IFN-γ test in Ireland, since April 2021, cattle that had an inconclusive result in the intradermal test were retested with the IFN-γ test; if positive, the animal was considered reactive and removed (52). In Spain, the IFN-γ test was used on animals older than 6 months, in parallel with the SIT and CIT, in herds with confirmed TB infection. The frequency of testing was established according to the prevalence in different areas and was specified in the National Eradication Program (37). In the United Kingdom, IFN-γ test has been used in certain infected herds with the aim of the rapid isolation and slaughter of infected and/or suspect animals to minimize TB spread (51).

In conclusion, the results obtained in the last few years of buffalo TB eradication programs in the Campania region and the experiences of other countries suggest that the following actions are required to achieve substantial improvements in the effectiveness of TB eradication programs: (i) an integrated approach between all the actors involved, also providing in the future the involvement of stakeholders and breeders; (ii) regular monitoring and evaluation of policies adopted; (iii) constant research activity aimed at overcoming diagnostic tool limits; (iv) implementation of biosecurity measures in risk areas; and (v) regular review of the strategies applied and rapid correction of any ineffective approaches.

To address the question, “Are We Heading Toward Eradication?” all epidemiological indicators and trend analyses indicate a decrease in prevalence and incidence, suggesting that the goal of bubaline tuberculosis eradication is within reach in Campania region.

## Data availability statement

The raw data supporting the conclusions of this article will be made available by the authors upon request.

## Author contributions

AM: Visualization, Writing – original draft, Writing – review & editing, Conceptualization, Data curation, Funding acquisition, Investigation, Methodology, Project administration, Resources, Supervision. MO: Data curation, Formal analysis, Methodology, Software, Supervision, Writing – original draft, Writing – review & editing. PM: Conceptualization, Data curation, Formal analysis, Investigation, Methodology, Supervision, Validation, Visualization, Writing – original draft, Writing – review & editing. NV: Conceptualization, Data curation, Formal analysis, Investigation, Methodology, Software, Supervision, Validation, Visualization, Writing – original draft, Writing – review & editing. AD: Formal analysis, Investigation, Writing – original draft, Writing – review & editing. RB: Data curation, Formal analysis, Methodology, Software, Supervision, Writing – original draft, Writing – review & editing. MF: Formal analysis, Investigation, Methodology, Writing – review & editing. PC: Formal analysis, Investigation, Methodology, Writing – review & editing. CS: Formal analysis, Investigation, Methodology, Writing – review & editing. GG: Writing – original draft, Supervision, Writing – review & editing. EC: Conceptualization, Funding acquisition, Investigation, Methodology, Resources, Supervision, Validation, Visualization, Writing – original draft, Writing – review & editing.

## Footnotes

^1^^1^Campania Region Executive Decree 236 of 14 October 2016. Procedures on the application of Community, national, regional and national legislation for the eradication of tuberculosis in cattle and buffaloes—amendment annexed to DD 226 of 3 October 2016.

^2^Campania Region Executive Decree 226 of 3 October 2016. Procedures on the application of Community, national, regional legislation for the eradication of tuberculosis in cattle and buffaloes.

^3^DGRC No. 207 of 20/05/2019 “Extraordinary plan for the control of Infectious Diseases of the Italian Mediterranean buffalo for the Campania Region” with its BRC/TBC/LEB annex.

^4^DGRC 104 of 08 March 2022 “Compulsory program of eradication from infectious diseases of bovine and buffalo species in Campania Region.”

^5^DD. 59 of 03/03/2017 concerning the ‘Approval of diagnostic procedures for the eradication of buffalo tuberculosis in Campania’.

^6^Presidential Decree of Campania Region of 04 August 2017, n.250 “Control and monitoring of the risks of spreading brucellosis and animal tuberculosis.”
