# Supplementary material for: A decade of tuberculosis eradication programs in the Mediterranean water buffalo (Bubalus bubalis) in South Italy: Are we heading toward eradication?
Source: Front Vet Sci. 2024 Jul 26;11:1405416. doi: 10.3389/fvets.2024.1405416 (PMC11310139; doi:10.3389/fvets.2024.1405416)
Supplement: Supplementary file 1 [file Data_Sheet_1.docx]

Supplementary Material

# Supplementary Figures and Tables

**Supplementary Table 1.** Campania Region TB Buffalo Culling 2012–2022

| Year | N. animals | N. positive animals cullings/ slaughtering | N. negative animals cullings/ slaughtering | N. animals cullings/ slaughtering | % cullings |
| --- | --- | --- | --- | --- | --- |
| 2012 | 291.994 | 868 | 6 | 874 | 0,30% |
| 2013 | 293.700 | 803 | 78 | 881 | 0,30% |
| 2014 | 296.133 | 855 | 349 | 1.204 | 0,41% |
| 2015 | 296.041 | 1.313 | 77 | 1.390 | 0,47% |
| 2016 | 300.565 | 259 | 124 | 383 | 0,13% |
| 2017 | 303.416 | 704 | 0 | 704 | 0,23% |
| 2018 | 316.152 | 5.896 | 854 | 6.750 | 2,14% |
| 2019 | 317.967 | 3.773 | 1.447 | 5.220 | 1,64% |
| 2020 | 320.688 | 2.749 | 1.743 | 4.492 | 1,40% |
| 2021 | 308.426 | 956 | 147 | 1.103 | 0,36% |
| 2022 | 302.386 | 494 | 0 | 498 | 0,16% |
| Total | 3.347.468 | 18.670 | 4.825 | 23.499 | 0,70% |

Data source: Vetinfo SIR 2012–2022

## Supplementary Figures

**Supplementary Figure 1**. Distribution of the number of buffaloes under the program and number of buffaloes tested per year (Data source SIR 2012-2022 – Vetinfo).


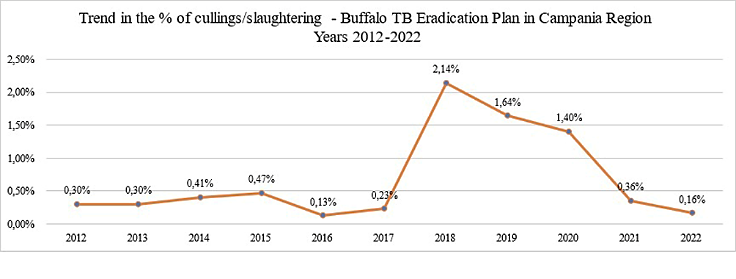


**Supplementary Figure 2.** Campania Region distribution of TB Buffaloes Culling per year (Data source: Vetinfo SIR 2012-2022).

**Supplementary Synoptic Table. Legislation and actions undertaken in the reference years 2012 – 2022.**

| **Legislation in-force data** | **Testing frequency** | **Antemortem diagnostic tests** | **Definition of suspected TB outbreak / Epidemiological investigation** | **Definition of TB outbreak** | **Disease-control measures** | **Use of the IFN-γ test** |
| --- | --- | --- | --- | --- | --- | --- |
| **DM 592/95**  14/06/1996 | Annually | **SIT:**  **CIT:** At the discretion of the veterinary service | In case of contact with infected animals  In case of inconclusive diagnostic tests | - Positive reaction to SIT/CIT  - Positive reaction to laboratory examinations | Inconclusive and/or positive animals must be isolated. Slaughter within 30 days of notification. Milk from positive animals shall be destroyed or used for animal feed after heat treatment. Milk produced by other buffaloes shall be heat-treated and used only for the manufacture of heat-treated milk or milk products. Prohibition of movement. Prohibition of natural mounting. Thorough cleaning and disinfection within 7 days of culling positive animals. Isolation of calves born from infected mothers with implementation of SIT. | Not provided |
| **DLvo 196/1999**  **09/07/1999** | Annually | **SIT**  **CIT** | Positive reaction to SIT  Suspected lesions at the slaughterhouse | - Isolation of *M.bovis.*  By decision of the competent authority:  - TB lesions at slaughterhouse  - Epidemiological correlations | Positive animals must be isolated and slaughter: if negative on post-mortem examinations, SIT is carried out on the entire herd and if negative the farm becomes OTF;  - Epidemiological investigation  - Cleaning and disinfection of animal enclosures, containers, equipment and tools used for animals | Not provided |
| **DD Campania n. 226/2016**  **DD Campania n. 236/2016**  03/10/2016 | OTF once a year by June 30 of the current year | **SIT**  **CIT**: In inconclusive and/or positive SIT animals after 42 days after SIT | Suspected epidemiological investigation. Inconclusive SIT/CIT in 1 or more animals. TB lesions at slaughterhouse. If until 30/06, controls have not been performed.  Epidemiological investigation within 2 days from suspected | - Epidemiological correlations  - For justified suspect of the presence of TB.  - Isolation of *M. bovis* | As set out in **DM 592/95, DLvo 196/1999** | Provided, together with CIT:  In case of 1 or more inconclusive and/or positive SIT animals after 42 days from SIT |
| **DGRC 207/2019**  20/05/2019 | OTF once a year.  In areas subject to risk (epidemiological situation / presence of repeated outbreaks), monitoring every six months. | **SIT**  **IFN-γ test** | Animals with a positive or inconclusive SIT are retested after 15 days with the IFN-γ test. If the farm had a TB outbreak, lesions at slaughter, or a previous suspension in the last 3 years, the qualification must be suspended, and positive animals are slaughtered.  If all animals test negative, on supplementary investigations the suspension is removed If at least one animal is IFN-γpositive, all SIT and/or IFN-γ positive animals must be slaughtered, and the herd's status remains suspended | Positive IFN-γ test and  - lesions at slaughterhouse;  - PCR positive;  - bacteriological positive. | As set out in **DM 592/95, DLvo 196/1999, DD 226/2016,** in addition:  - Positive animals are moved to slaughterhouse upon notice Area B of the concerned slaughterhouse at least 48 hours in advance to carry out the expected sampling.  - The housings, containers, equipment, and utensils used for the animals must be thoroughly cleaned and disinfected. All vehicles used to move animals from an infected herd must be cleaned and disinfected after each transport.  - Surveillance of all sheep/goats on the farm with SIT. | The region, upon the proposal of the responsible of Local Official Veterinary Service, may authorize the use of IFN-γ test for the rapid extinction of outbreaks |
| **DGRC 104/2022**  January 2022 | OTF once a year | **SIT**  **IFN-γ test** | Even a single animal with a positive SIT screening test.  In case of negative postmortem, epidemiological investigation, and negative IFN-γ test (tested after 42 days and within 60 days from the last SIT), the whole herd reacquires the status “Indemnified”. | - SIT/ IFN-γ test positive/inconclusive and  - Slaughterhouse lesions  - PCR positive  - Bacteriological positive | As set out in **DM 592/95**, **DLvo 196/1999**, **DD 226/2016**, **DGRC 207/2019** in addition:  - Prevent access to outsiders-staff, animals of other species, including wild animals. | **In outbreaks or suspected herds:**  in alternation with the SIT; in case of positive SIT and negative postmortem diagnostic test/ **In OTF:** presence of TB lesions at slaughterhouse; inconclusive SIT; movements; suspect for epidemiological correlation with established outbreak. **Any other herds** on discretion of the official veterinary service to accelerate local TB eradication program. |
